# Supplementary material for: Yearly Incidence of Stroke and Bleeding in Atrial Fibrillation with Concomitant Hyperthyroidism: A National Discharge Database Study
Source: J Clin Med. 2022 Feb 28;11(5):1342. doi: 10.3390/jcm11051342 (PMC8911027; doi:10.3390/jcm11051342)
Supplement: Supplementary file 1 [file jcm-11-01342-s001.zip › jcm-1593239-supplementary.pdf]

Supplementary tables and figure legends for :

Yearly incidence of stroke and bleeding in atrial fibrillation with concomitant hyperthyroidism: A national discharge database study

Juqian ZHANG, Arnaud BISSON, Grégoire FAUCHIER, Alexandre BODIN, Julien HERBERT, Pierre Henri DUCLUZEAU, Gregory Y.H. LIP*, Laurent FAUCHIER

Supplementary Table S1. Baseline characteristics of AF patients admitted to French hospitals (2010-2019) according to the presence and absence of concomitant hyperthyroidism in men (1a) and women (1b), respectively.

1a. Baseline characteristics of men included in the study:

	**No Hyperthyroidism (n=1269048)**	**Hyperthyroidism (n=12245)**	**p**	**Total (n=1281293)**
**Age (years), mean±SD**	74.4±12.3	73.4±12.6	<0.0001	74.3±12.3
**Sex (male), n (%)**	1269048 (100.0)	12245 (100.0)	-	1281293 (100.0)
**CHA2DS2VASc score, mean±SD**	2.9±1.6	2.8±1.6	0.05	2.9±1.6
**HASBLED score, mean±SD**	2.4±1.3	2.4±1.3	0.15	2.4±1.3
**Charlson comorbidity index, mean±SD**	3.6±3.0	3.8±3.0	<0.0001	3.6±3.0
**Frailty index, mean±SD**	8.4±8.8	9.7±9.3	<0.0001	8.4±8.8
**Hypertension, n (%)**	723226 (57.0)	6894 (56.3)	0.13	730120 (57.0)
**Diabetes mellitus, n (%)**	288790 (22.8)	2873 (23.5)	0.06	291663 (22.8)
**Heart failure with congestion, n (%)**	459038 (36.2)	5125 (41.9)	<0.0001	464163 (36.2)
**History of pulmonary edema, n (%)**	34161 (2.7)	325 (2.7)	0.8	34486 (2.7)
**Mitral regurgitation, n (%)**	69322 (5.5)	782 (6.4)	<0.0001	70104 (5.5)
**Aortic regurgitation, n (%)**	32610 (2.6)	285 (2.3)	0.09	32895 (2.6)
**Aortic stenosis, n (%)**	78004 (6.1)	608 (5.0)	<0.0001	78612 (6.1)
**Previous endocarditis, n (%)**	7263 (0.6)	49 (0.4)	0.01	7312 (0.6)
**Dilated cardiomyopathy, n (%)**	103776 (8.2)	1338 (10.9)	<0.0001	105114 (8.2)
**Coronary artery disease, n (%)**	379467 (29.9)	3491 (28.5)	0.001	382958 (29.9)
**Previous MI, n (%)**	72306 (5.7)	617 (5.0)	0.002	72923 (5.7)
**Previous PCI, n (%)**	69346 (5.5)	577 (4.7)	0.0003	69923 (5.5)
**Previous CABG, n (%)**	63226 (5.0)	514 (4.2)	0.0001	63740 (5.0)
**Vascular disease, n (%)**	275172 (21.7)	2612 (21.3)	0.35	277784 (21.7)
**Previous pacemaker or ICD, n (%)**	60301 (4.8)	606 (4.9)	0.31	60907 (4.8)
**Ischemic stroke, n (%)**	80099 (6.3)	635 (5.2)	<0.0001	80734 (6.3)
**Intracranial bleeding, n (%)**	27969 (2.2)	242 (2.0)	0.09	28211 (2.2)
**Smoker, n (%)**	127338 (10.0)	1420 (11.6)	<0.0001	128758 (10.0)
**Dyslipidaemia, n (%)**	300815 (23.7)	2686 (21.9)	<0.0001	303501 (23.7)
**Obesity, n (%)**	187577 (14.8)	1714 (14.0)	0.02	189291 (14.8)
**Alcohol related diagnoses, n (%)**	96808 (7.6)	882 (7.2)	0.08	97690 (7.6)
**Abnormal renal function, n (%)**	87379 (6.9)	961 (7.8)	<0.0001	88340 (6.9)
**Lung disease, n (%)**	236597 (18.6)	2605 (21.3)	<0.0001	239202 (18.7)
**Sleep apnoea syndrome, n (%)**	90273 (7.1)	832 (6.8)	0.17	91105 (7.1)
**COPD, n (%)**	153906 (12.1)	1789 (14.6)	<0.0001	155695 (12.2)
**Liver disease, n (%)**	54959 (4.3)	536 (4.4)	0.8	55495 (4.3)
**History of hypothyroidism, n (%)**	43027 (3.4)	609 (5.0)	<0.0001	43636 (3.4)
**Inflammatory disease, n (%)**	72825 (5.7)	746 (6.1)	0.09	73571 (5.7)
**Anaemia, n (%)**	204378 (16.1)	2129 (17.4)	0.0001	206507 (16.1)
**Previous cancer, n (%)**	266767 (21.0)	2452 (20.0)	0.01	269219 (21.0)

Values are n (%) or mean±SD. CABG: coronary artery bypass graft; COPD: chronic obstructive pulmonary disease; MI: myocardial infarction; ICD: implantable cardioverter defibrillator; PCI: percutaneous coronary intervention; SD: standard deviation.

1b. Baseline characteristics of women included in the study:

	No Hyperthyroidism (n=1119639)	Hyperthyroidism (n=20155)	p	Total (n=1139794)
**Age (years), mean±SD**	80.4±10.9	79.2±11.9	<0.0001	80.4±11.0
**Sex (male), n (%)**	0 (0.0)	0 (0.0)	-	0 (0.0)
**CHA2DS2VASc score, mean±SD**	4.1±1.4	4.1±1.4	<0.0001	4.1±1.4
**HASBLED score, mean±SD**	2.4±1.1	2.3±1.1	<0.0001	2.4±1.1
**Charlson comorbidity index, mean±SD**	3.2±2.6	3.2±2.5	0.04	3.2±2.6
**Frailty index, mean±SD**	10.2±9.4	10.9±9.5	<0.0001	10.2±9.4
**Hypertension, n (%)**	705035 (63.0)	12602 (62.5)	0.2	717637 (63.0)
**Diabetes mellitus, n (%)**	209782 (18.7)	3833 (19.0)	0.31	213615 (18.7)
**Heart failure with congestion, n (%)**	402535 (36.0)	8109 (40.2)	<0.0001	410644 (36.0)
**History of pulmonary edema, n (%)**	24915 (2.2)	400 (2.0)	0.02	25315 (2.2)
**Mitral regurgitation, n (%)**	70174 (6.3)	1398 (6.9)	0.0001	71572 (6.3)
**Aortic regurgitation, n (%)**	25720 (2.3)	522 (2.6)	0.01	26242 (2.3)
**Aortic stenosis, n (%)**	68764 (6.1)	1134 (5.6)	0.003	69898 (6.1)
**Previous endocarditis, n (%)**	3274 (0.3)	61 (0.3)	0.79	3335 (0.3)
**Dilated cardiomyopathy, n (%)**	60850 (5.4)	1382 (6.9)	<0.0001	62232 (5.5)
**Coronary artery disease, n (%)**	205474 (18.4)	3307 (16.4)	<0.0001	208781 (18.3)
**Previous MI, n (%)**	44530 (4.0)	737 (3.7)	0.02	45267 (4.0)
**Previous PCI, n (%)**	26005 (2.3)	401 (2.0)	0.002	26406 (2.3)
**Previous CABG, n (%)**	15227 (1.4)	199 (1.0)	<0.0001	15426 (1.4)
**Vascular disease, n (%)**	144243 (12.9)	2391 (11.9)	<0.0001	146634 (12.9)
**Previous pacemaker or ICD, n (%)**	32034 (2.9)	392 (1.9)	<0.0001	32426 (2.8)
**Ischemic stroke, n (%)**	90184 (8.1)	1318 (6.5)	<0.0001	91502 (8.0)
**Intracranial bleeding, n (%)**	23527 (2.1)	340 (1.7)	<0.0001	23867 (2.1)
**Smoker, n (%)**	31815 (2.8)	716 (3.6)	<0.0001	32531 (2.9)
**Dyslipidaemia, n (%)**	212917 (19.0)	3421 (17.0)	<0.0001	216338 (19.0)
**Obesity, n (%)**	152002 (13.6)	2460 (12.2)	<0.0001	154462 (13.6)
**Alcohol related diagnoses, n (%)**	23433 (2.1)	326 (1.6)	<0.0001	23759 (2.1)
**Abnormal renal function, n (%)**	75171 (6.7)	1304 (6.5)	0.17	76475 (6.7)
**Lung disease, n (%)**	156990 (14.0)	2853 (14.2)	0.59	159843 (14.0)
**Sleep apnoea syndrome, n (%)**	34396 (3.1)	491 (2.4)	<0.0001	34887 (3.1)
**COPD, n (%)**	69337 (6.2)	1274 (6.3)	0.45	70611 (6.2)
**Liver disease, n (%)**	31827 (2.8)	569 (2.8)	0.87	32396 (2.8)
**History of hypothyroidism, n (%)**	125424 (11.2)	1941 (9.6)	<0.0001	127365 (11.2)
**Inflammatory disease, n (%)**	68979 (6.2)	1208 (6.0)	0.33	70187 (6.2)
**Anaemia, n (%)**	199723 (17.8)	3518 (17.5)	0.16	203241 (17.8)
**Previous cancer, n (%)**	153310 (13.7)	2391 (11.9)	<0.0001	155701 (13.7)

Values are n (%) or mean±SD. CABG: coronary artery bypass graft; COPD: chronic obstructive pulmonary disease; MI: myocardial infarction; ICD: implantable cardioverter defibrillator; PCI: percutaneous coronary intervention; SD: standard deviation.

Supplementary Table S2. Yearly incidence of stroke in AF patients stratified by sex, presence or absence of concomitant hyperthyroidism and the CHA_2DS_2VASc score.

**CHA_2DS_2VASc score**	**All patients, hyperthyroidism**	**All patients, no hyperthyroidism**	**Men, hyperthyroidism**	**Men, no hyperthyroidism**	**Women, hyperthyroidism**	**Women, no hyperthyroidism**
Whole follow-up:						
0	0.5 (0.3-0.9)	0.7 (0.6-0.7)	0.5 (0.3-0.9)	0.7 (0.6-0.7)	-	-
1	0.8 (0.6-1.1)	0.9 (0.9-0.9)	0.7 (0.5-1.0)	1.0 (1.0-1.0)	0.9 (0.6-1.4)	0.6 (0.6-0.7)
2	1.4 (1.2-1.6)	1.4 (1.4-1.5)	1.5 (1.3-1.9)	1.6 (1.5-1.6)	1.1 (0.8-1.5)	1.0 (1.0-1.0)
3	2.2 (2.0-2.4)	2.0 (2.0-2.1)	1.9 (1.6-2.3)	2.0 (2.0-2.0)	2.4 (2.1-2.8)	2.1 (2.0-2.1)
4	3.2 (2.9-3.4)	2.6 (2.6-2.6)	2.9 (2.4-3.4)	2.6 (2.5-2.6)	3.3 (2.9-3.6)	2.6 (2.6-2.7)
5	3.7 (3.3-4.1)	3.4 (3.4-3.5)	4.3 (3.4-5.3)	3.5 (3.5-3.6)	3.5 (3.1-4.0)	3.4 (3.3-3.4)
6	5.1 (4.4-5.9)	5.3 (5.2-5.3)	4.7 (3.3-6.7)	5.8 (5.6-5.9)	5.2 (4.5-6.1)	5.1 (5.0-5.2)
7	7.5 (5.9-9.5)	8.0 (7.7-8.2)	13.5 (7.3-25.2)	10.2 (9.6-10.8)	6.9 (5.4-9.0)	7.5 (7.2-7.7)
8	16.6 (11.2-24.6)	11.3 (10.7-11.9)	-	10.3 (9.1-11.5)	14.0 (8.9-21.9)	11.6 (10.9-12.3)
9	15.9 (4.0-63.8)	13.2 (11.6-15.0)	-	-	15.9 (4.0-63.8)	13.2 (11.6-15.0)
Total	2.6 (2.5-2.8)	2.3 (2.3-2.4)	2.0 (1.8-2.2)	2.0 (2.0-2.0)	3.1 (2.9-3.3)	2.8 (2.8-2.8)

Supplementary Table S3. Hazard ratios for the association between hyperthyroidism and ischaemic stroke during 1) whole follow-up, 2) first year of follow-up after concomitant diagnoses of AF and hyperthyroidism, and 3) beyond the first year of follow-up.

	**Whole FU**	**Day 1-365**	**After Day 365**
CHA_2DS_2VASc score, all patients			
0-1	0.851 (0.663-1.093)	0.992 (0.638-1.542)	0.797 (0.588-1.081)
2	0.957 (0.806-1.137)	1.136 (0.862-1.498)	0.871 (0.700-1.084)
3	1.074 (0.961-1.201)	1.106 (0.921-1.328)	1.056 (0.918-1.216)
4	1.206 (1.105-1.317)	1.318 (1.154-1.505)	1.131 (1.006-1.272)
5	1.068 (0.963-1.183)	1.144 (0.991-1.321)	0.999 (0.863-1.156)
6-9	0.990 (0.881-1.113)	1.035 (0.893-1.200)	0.923 (0.763-1.118)
Total	1.114 (1.061-1.169)	1.203 (1.120-1.291)	1.047 (0.980-1.118)
CHA_2DS_2VASc score, Men			
0-1	0.728 (0.535-0.990)	0.907 (0.536-1.535)	0.661 (0.452-0.965)
2	0.989 (0.806-1.213)	1.125 (0.806-1.569)	0.921 (0.711-1.193)
3	0.951 (0.794-1.139)	1.131 (0.865-1.478)	0.840 (0.658-1.072)
4	1.120 (0.943-1.329)	1.253 (0.986-1.591)	1.006 (0.787-1.287)
5-8	1.094 (0.916-1.306)	1.227 (0.988-1.523)	0.893 (0.654-1.219)
Total	0.986 (0.903-1.076)	1.159 (1.024-1.312)	0.857 (0.758-0.970)
CHA_2DS_2VASc score, Women			
1-2	1.180 (0.913-1.524)	1.500 (0.981-2.294)	1.050 (0.761-1.448)
3	1.157 (1.004-1.334)	1.100 (0.855-1.414)	1.187 (0.999-1.410)
4	1.238 (1.118-1.372)	1.393 (1.187-1.634)	1.149 (1.005-1.313)
5	1.046 (0.932-1.175)	1.140 (0.965-1.347)	0.972 (0.828-1.141)
6-9	1.007 (0.887-1.144)	1.026 (0.870-1.210)	0.980 (0.802-1.198)
Total	1.100 (1.038-1.166)	1.150 (1.054-1.254)	1.063 (0.983-1.149)

Hazard ratios are for hyperthyroidism vs. no hyperthyroidism. FU: follow-up.
